# A clinical case analysis of a misdiagnosis as AS, actually DISH: A case report

**DOI:** 10.1097/MD.0000000000044121

**Published:** 2025-08-22

**Authors:** Shiwei Yuan, Xuanhua Yu, Xiuqing Luo, Siyu Liu

**Affiliations:** a Department of Acupuncture, Longyan Hospital of Xiamen University, School of Medicine, Xiamen University, Longyan, China; b Department of Rheumatology, People's Hospital Affiliated to Fujian University of Traditional Chinese Medicine, Fuzhou, China.

**Keywords:** AS, clinical case, DISH, misdiagnosis

## Abstract

**Rationale:**

Axial spondyloarthritis (axSpA) and diffuse idiopathic skeletal hyperostosis (DISH) exhibit substantial overlap in clinical symptoms and imaging features, frequently resulting in diagnostic errors. This case study delves into a patient initially misdiagnosed with axSpA but later confirmed to have DISH, aiming to dissect the root causes of misdiagnosis, identify critical diagnostic criteria, and devise strategies to enhance diagnostic accuracy between these 2 conditions.

**Patient concerns:**

The patient initially presented with chronic axial pain and stiffness, symptoms commonly associated with axSpA, leading to an initial misdiagnosis. This misdiagnosis raised concerns about the effectiveness of the prescribed treatment regimen and potential long-term implications for the patient’s health.

**Diagnoses:**

The initial diagnosis of axSpA was based on the patient’s symptoms and preliminary imaging results. However, through further in-depth investigation, including a comprehensive collection of clinical manifestations, more detailed imaging analysis, and multidisciplinary consultation, the final diagnosis was revised to DISH.

**Interventions:**

To rectify the misdiagnosis, strategies such as a more thorough gathering of patients’ symptoms and signs, rational utilization of multiple auxiliary examinations (including advanced imaging techniques and laboratory tests), and collaboration with multiple disciplines (such as rheumatology, radiology, and orthopedics) were implemented.

**Outcomes:**

These interventions led to the accurate diagnosis of DISH, enabling the development of an appropriate treatment plan tailored to the patient’s actual condition. As a result, the patient’s symptoms were better managed, and the prognosis was improved.

**Lessons:**

This case underscores the importance of recognizing the limitations of initial diagnostic approaches, overcoming cognitive biases and fixed thinking patterns among physicians, and the value of comprehensive, multidisciplinary evaluations. It provides valuable insights for clinicians to enhance their ability to distinguish between axSpA and DISH, thereby reducing the likelihood of misdiagnosis and improving overall diagnostic and treatment quality.

## 1. Introduction

Diffuse idiopathic skeletal hyperostosis (DISH) is a relatively common disease of the skeletal system that has received widespread attention in recent years.^[1,2]^ The main feature of DISH is heterotopic ossification of the spine and peripheral bones, usually manifested as calcification and ossification of the anterolateral ligaments of the spine, leading to the formation of bone bridges between vertebral bodies.^[1–3]^ Although it is not uncommon in clinical practice, the insufficient understanding of its pathogenesis and the diversity of disease manifestations pose many challenges to accurate diagnosis and effective treatment.^[1]^

Current studies have shown that the pathogenesis of DISH is related to various factors, including genetic, metabolic, vascular and mechanical factors.^[2]^ However, the interaction between these factors and the specific pathogenic mechanisms have not been fully clarified, which to some extent limits the development of precise diagnosis and treatment strategies for DISH. In terms of diagnosis, although the criteria proposed by Resnick and Niwayama are widely used in clinical practice, these criteria are mainly applicable to the advanced stage of the disease and have limitations in the diagnosis of early DISH, resulting in some patients failing to receive timely diagnosis and treatment.^[4]^

It is worth noting that misdiagnosis of DISH is relatively common.^[5]^ Due to the similarity of clinical manifestations of DISH to some other spinal diseases, such as axSpA and degenerative osteoarthritis, clinicians are prone to misdiagnosis during the diagnostic process.^[6]^ Such misdiagnosis may lead to patients receiving inappropriate treatment, which not only delays the condition but also may cause a series of potential clinical consequences, such as disease progression and increased risk of complications, seriously affecting the quality of life and prognosis of patients.

There are many diseases related to DISH, and each has its unique typical symptoms. For example, axSpA tends to occur in young and middle-aged men, with main symptoms of inflammatory low back pain, which may be accompanied by swelling and pain of peripheral joints and extra-articular manifestations (such as iritis, chronic inflammatory bowel disease or skin lesions, etc). Its sacroiliac joint lesions are mainly erosion and ulceration, and the bamboo-like changes of the spine usually appear in the advanced stage of the disease.^[7]^ Degenerative osteoarthritis, on the other hand, mainly involves articular cartilage and bone, manifested as joint pain, swelling, deformity and limited movement, which is different from the ossification characteristics of DISH. Accurate identification of the typical symptoms of these diseases is crucial for the differential diagnosis of DISH.

Among numerous DISH cases, some special cases have unique research value. For example, some cases may be complicated with other rare diseases, or present atypia in clinical manifestations and imaging features. These special cases provide valuable clues for in-depth understanding of the pathogenesis, clinical characteristics and treatment strategies of DISH.^[8]^ Through the study of these special or representative cases, we can further enrich the understanding of DISH, provide more targeted guidance for clinical practice, and help improve the overall diagnosis and treatment level of DISH. Now, a case misdiagnosed as axSpA but actually DISH is analyzed, and written informed consent has been obtained from the patient in this article.

## 2. Case presentation

### 2.1. Patient history

A 67-year-old Chinese male patient was admitted to the hospital with the main complaint of “lumbosacral soreness and limited mobility for more than 30 years, aggravated for 1 month.” The patient had recurrent lumbosacral soreness for over 30 years. More than 10 years ago, the soreness in the lumbosacral region worsened, and he visited a hospital in our city, where he was diagnosed with “possible axial spondyloarthritis.” After treatment with nonsteroidal anti-inflammatory drugs, his symptoms were relieved. Later, the lumbosacral soreness recurred, so he went to the hospital in our city again. The examination showed HLA-B-27 (−), and the sacroiliac joint MRI showed no obvious bone marrow edema, considering “seronegative axial spondyloarthritis.” His symptoms were slightly relieved after treatment with nonsteroidal anti-inflammatory drugs and physical therapy. Subsequently, he was hospitalized in the orthopedics department of our hospital due to lumbosacral soreness with limited mobility, and was diagnosed with axSpA again. The patient refused biological agents and continued to receive physical therapy, anti-inflammatory and analgesic drugs. Now, the patient visited our department again due to lumbosacral pain.

### 2.2. Physical examination, laboratory and imaging findings

**Figure 1.**
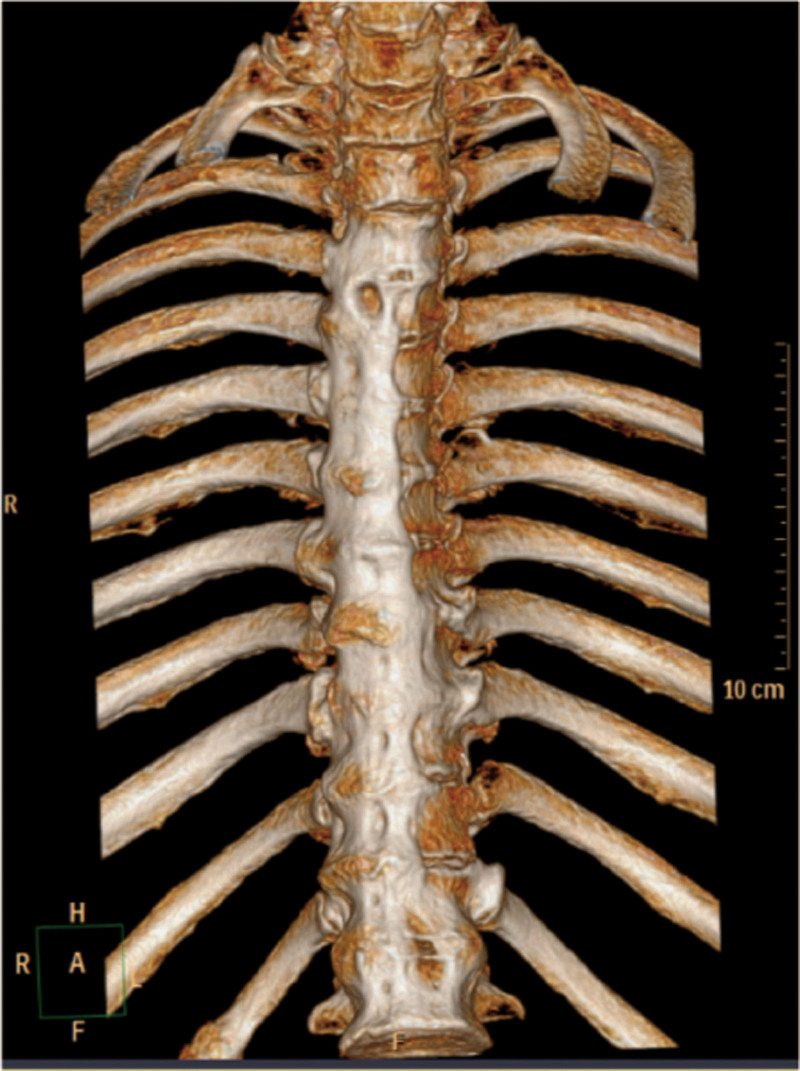
CT 3D imaging of thoracic vertebra: it can be seen that there are bright connections formed by calcification of the anterior longitudinal ligaments of multiple segments.

**Figure 2.**
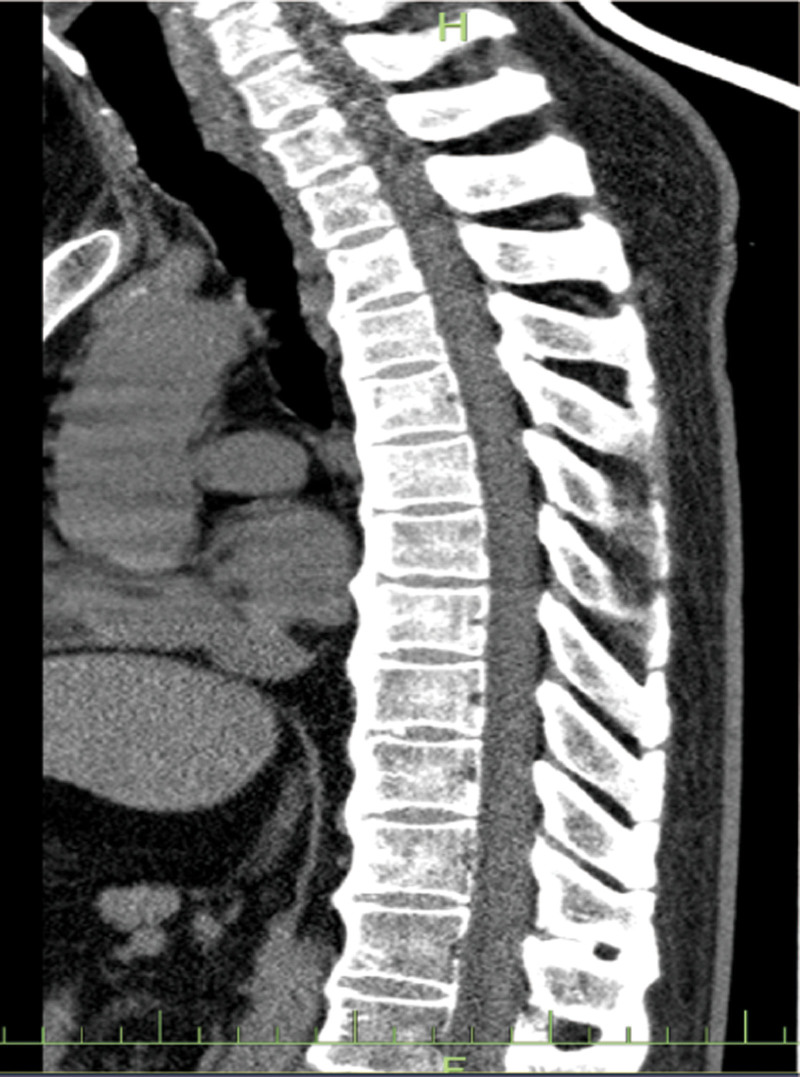
Sagittal position of thoracic vertebra: it can be seen that the changes of bone Bridges formed by calcification of the anterior longitudinal ligaments of multiple segments.

**Figure 3.**
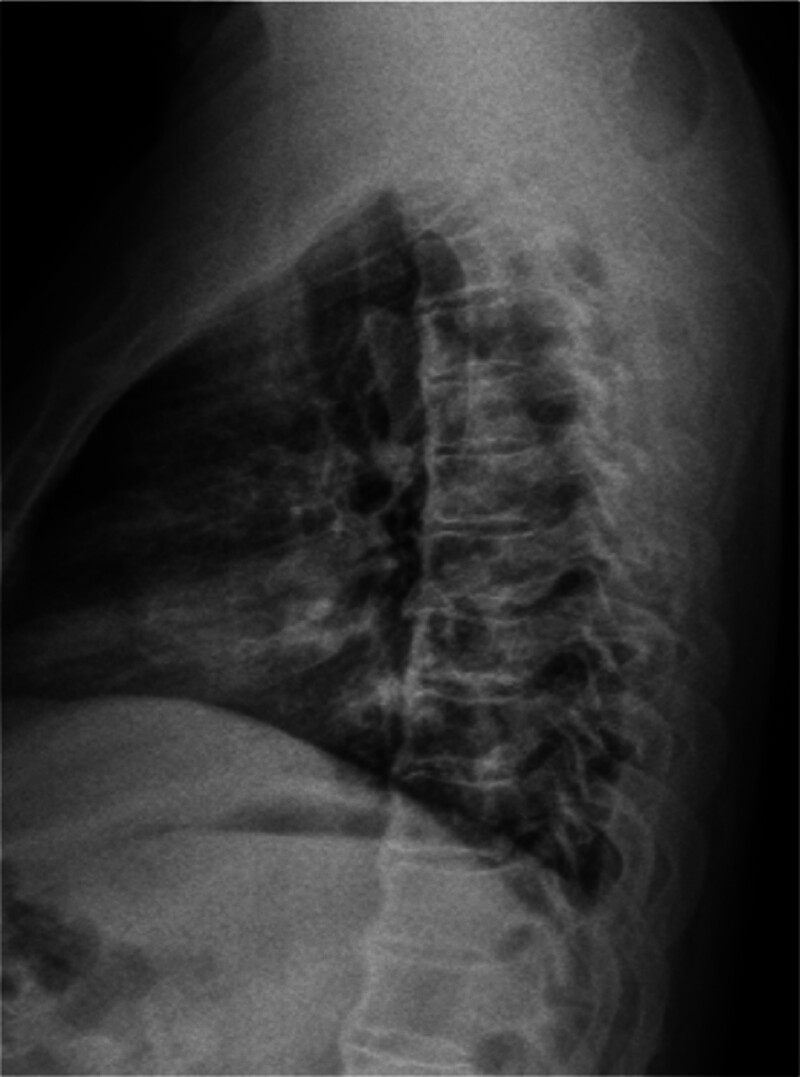
Lateral plain film of thoracic vertebra: it can be seen that there is osteoporosis and bone degeneration.

**Figure 4.**
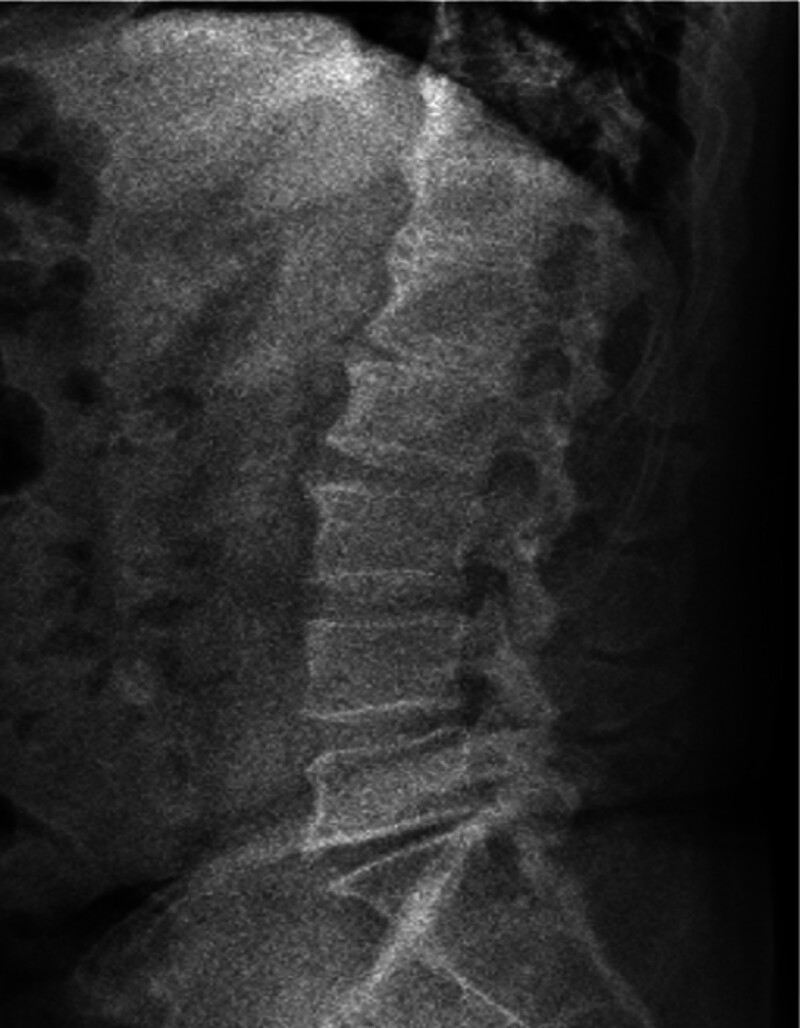
Lateral plain film of lumbar spine: it can be seen that there is osteoporosis and bone degeneration.

**Figure 5.**
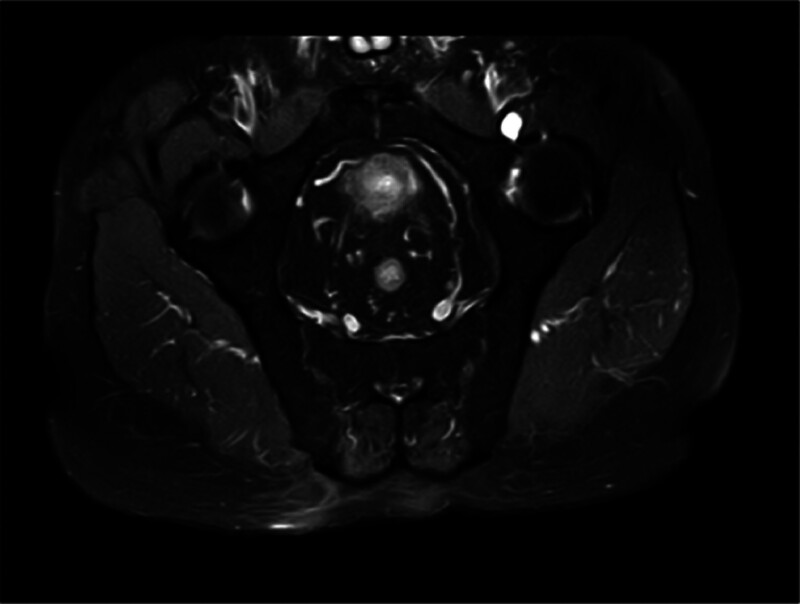
Sacroiliac joint magnetic resonance: no obvious abnormal signals of the sacroiliac joint or roughness of the sacroiliac joint were observed.

**Figure 6.**
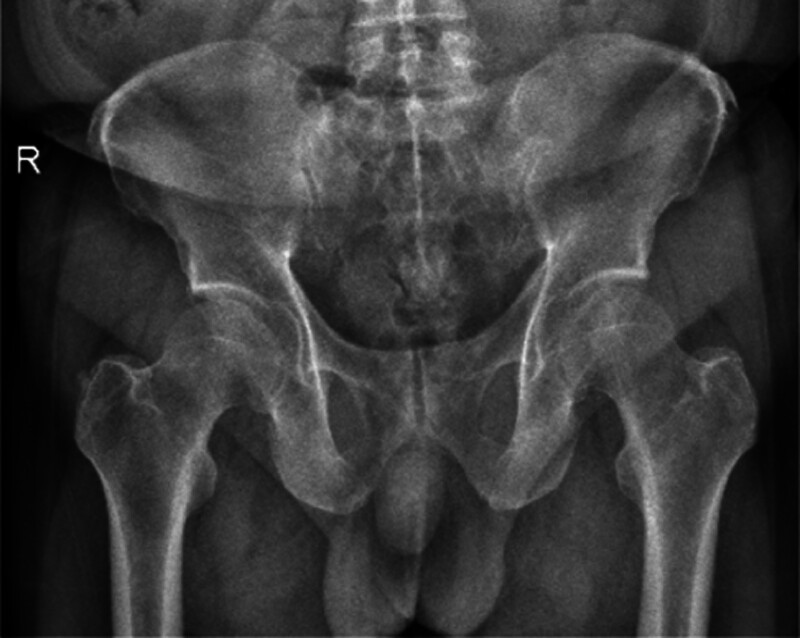
Pelvic orthograph: no obvious fusion lesion of the sacroiliac joint was observed.

The patient’s spine was in the middle, with the cervical spine in a forward flexed position, and the neck movement was acceptable. The lumbar and back movement was slightly limited. The Schober test was 7cm. When standing and lateral bending at 20 degrees, the distance from the apex to the iliac crest increased by about 3 to 4cm compared with the neutral position. The neck, waist and back muscles were tense. There was tenderness between the spinous processes of T10-L5 and on both sides of the spinous processes, tenderness in the bilateral sacroiliac joint space, negative percussion pain, and tenderness at the sensitive points of the bilateral piriformis and gluteus medius muscles. The bilateral straight leg raising test at 70 degrees was negative, the bilateral piriformis tension test was negative, the bilateral “4” sign test was positive, and the neurological examination was normal. CRP: normal; ESR: 21.4mm/h. Three-dimensional CT imaging of the thoracic vertebrae (Fig. 1), thoracic CT (Fig. 2): Degenerative changes of the thoracic vertebrae. Thoracic spine anteroposterior and lateral films (Fig. 3), lumbar spine anteroposterior and lateral films (Fig. 4): Degenerative changes of the thoracolumbar spine. MRI of the sacroiliac joint (Fig. 5): No obvious bone marrow edema was observed. Pelvic plain film: No obvious abnormalities were found (Fig. 6). Dual-energy X-ray bone mineral density: osteoporosis of the left femur (*t* value: −2.8).

After multiple consultations with rheumatology and immunology experts inside and outside the hospital, based on the patient’s clinical manifestations and spinal imaging findings, the final diagnosis of this case was DISH, excluding axSpA. It was suggested to adopt the treatment method for osteoarthritis, with “anti-inflammatory and analgesic, improving bone metabolism, anti-osteoporosis” and other treatments. Therefore, he was given celecoxib, calcium carbonate, alfacalcidol and other treatments for 7 days, and the patient’s symptoms were relieved and discharged.

## 3. Results

This study conducted a detailed analysis of a 67-year-old male patient initially misdiagnosed as axSpA but ultimately confirmed as DISH, yielding the following key results: definitive diagnosis through multidisciplinary collaboration. Over 30 years, the patient was repeatedly misdiagnosed as axSpA due to lumbosacral pain, limited mobility, and ambiguous imaging findings. A subsequent multidisciplinary consultation involving rheumatology experts reevaluated the clinical data, leading to a final diagnosis of DISH based on the integration of the following information. Clinical features: absence of typical axSpA manifestations, such as noninflammatory low back pain (morning stiffness lasting < 30 minutes with no improvement after activity), negative HLA-B-27, and normal CRP. Imaging evidence: X-rays and CT revealed thoracolumbar degenerative changes with anterior longitudinal ligament ossification (consistent with DISH characteristics), while MRI showed no sacroiliac joint inflammation (ruling out axSpA). Laboratory tests: Mildly elevated ESR (21.4 mm/h) without the typical systemic inflammatory marker abnormalities seen in axSpA.

Efficacy of DISH-specific treatment: after diagnosis correction, the patient received DISH-targeted therapy, including anti-inflammatory analgesics (celecoxib), bone metabolism modulators (calcium carbonate), and anti-osteoporotic drugs (alfacalcidol). Significant symptom relief was observed within 7 days, with reduced lumbosacral pain and improved mobility, confirming the effectiveness of this treatment regimen for DISH. Key factors contributing to misdiagnosis: analysis identified 2 main causes of misdiagnosis: overlapping clinical manifestations: Shared symptoms between DISH and axSpA (e.g., spinal pain, limited mobility) led to initial diagnostic bias. Limitations in imaging interpretation: Spinal calcifications observed on early X-rays were misinterpreted as degenerative changes or axSpA-related “bamboo spine,” failing to recognize DISH-specific features (e.g., extensive anterior longitudinal ligament ossification involving ≥ 4 consecutive vertebral bodies). Clinical insights for differential diagnosis:The case highlighted critical lessons for distinguishing DISH from axSpA:DISH predominantly affects individuals over 50 years of age, with no systemic inflammatory manifestations (normal HLA-B-27, mildly elevated or normal inflammatory markers). DISH is radiologically characterized by anterior longitudinal ligament ossification, whereas axSpA is defined by sacroiliitis and syndesmophyte formation (axial fusion). Multidisciplinary collaboration and comprehensive assessment of clinical, laboratory, and imaging data are pivotal to avoiding misdiagnosis.

These results further confirm that DISH should be included in the differential diagnosis for patients with spinal symptoms, particularly when axSpA-specific markers are absent. They also emphasize the value of targeted treatment based on accurate diagnosis.

## 4. Discussion

Three widely recognized radiological diagnostic criteria exist for DISH: those proposed by Resnick and Niwayama, and Julkunen and Utsinger.^[9,10]^ The most critical imaging indicator is the presence of ossification in at least 4 adjacent vertebrae. Due to the similarity of its clinical symptoms to those of other diseases, DISH is often misdiagnosed. In this case, the patient underwent X-ray, CT, MRI, and bone density examinations, with imaging findings playing a pivotal role in differential diagnosis:Thoracolumbar X-rays:showed “degenerative changes,” primarily manifested as vertebral edge hyperosteogeny, narrowed intervertebral spaces, and calcification of the anterolateral spinal ligaments. While partially overlapping with typical DISH imaging features (extensive anterior longitudinal ligament ossification and bone bridge formation between vertebral bodies), the mild early-stage ossification was easily misinterpreted as ordinary degenerative changes. Three-dimensional CT of the thoracic spine:further confirmed “degenerative changes” and revealed band-like calcification of the anterior longitudinal ligament involving multiple (>4) vertebrae, consistent with DISH diagnostic criteria (“ossification of at least 4 consecutive vertebral bodies”). No characteristic axSpA features were observed, such as sacroiliac joint erosion, sclerosis, or spinal syndesmophyte formation. Sacroiliac joint MRI: showed “no obvious inflammation,” i.e., absence of bone marrow edema, cartilage destruction, or joint space narrowing – typical inflammatory changes of axSpA. This directly ruled out the key diagnostic criterion for axSpA (sacroiliitis is a hallmark lesion of axSpA). Dual-energy X-ray bone densitometry: indicated “osteoporosis of the left femur (T-score: −2.8),” correlated with metabolic abnormalities (e.g., bone density disorders) commonly associated with DISH. In contrast, osteoporosis in axSpA is typically linked to chronic inflammation, and the lack of obvious inflammatory evidence in this case further supported a metabolic association with DISH.

The initial misdiagnosis as axSpA likely stemmed from the following contradictions in clinical judgment: partial overlap of symptoms and imaging findings: The patient’s lumbosacral pain, limited mobility, and spinal ligament calcifications on X-ray/CT resembled the late-stage “bamboo spine” ossification of axSpA. Ambiguous application of diagnostic criteria: early gender-recognition of DISH led to failure in strictly applying the Resnick criteria (≥4 consecutive vertebral body ossification). Spinal calcifications were vaguely categorized as “degenerative changes or axSpA-related ossification,” while the axSpA diagnosis was retained due to symptom similarity, creating an overlapping label. Misleading laboratory tests: The patient’s mildly elevated ESR (21.4 mm/h), though with normal CRP, was potentially misinterpreted by some clinicians as evidence of inflammatory activity, further supporting axSpA.

This, combined with imaging findings of ossification, led to a tentative diagnosis of axSpA. Key misleading factors identified in the analysis include: similarity of clinical manifestations: DISH most commonly presents with spinal and pelvic pain, limited mobility, and often lumbar/cervical ossification. These symptoms overlap significantly with those of rheumatological diseases like axSpA and degenerative joint disease, increasing the risk of misdiagnosis – particularly for atypical cases. In this case, the patient’s persistent lumbosacral pain, limited mobility, and reduced spinal flexibility mirrored early axSpA symptoms, leading clinicians to overlook other differential diagnoses and erroneously confirm axSpA based on symptoms, clinical presentation, and auxiliary tests. The presence of spinal calcifications on imaging further distracted from excluding DISH. Misinterpretation of tests and limitations of imaging diagnosis: Imaging modalities (especially X-ray, CT, or MRI) are critical for DISH diagnosis, but their findings may overlap with other spinal diseases (e.g., axSpA, degenerative spinal disease). In early-stage DISH, spinal ossification and ligament calcification may be subtle or masked by other pathologies. In this case, thoracic paravertebral soft tissue calcifications on X-ray were misattributed to ordinary degenerative spinal changes rather than prompting further investigation for DISH.

As a chronic inflammatory disease, axial spondyloarthritis (axSpA) is closely associated with immune-mediated inflammatory responses. Patients often present with elevated acute-phase reactant proteins such as C-reactive protein (CRP), and there is a certain correlation between disease activity and CRP levels. In contrast, DISH is characterized primarily by heterotopic ossification of the spine and peripheral skeleton (ligament calcification and bone bridge formation), which is essentially a noninflammatory condition and typically does not cause significant elevation of CRP. In this case, repeated examinations of the patient showed normal CRP levels, with only a mild elevation of erythrocyte sedimentation rate (ESR) at 21.4 mm/h. This result is inconsistent with the typical inflammatory marker profile of axSpA but aligns with the noninflammatory nature of DISH, serving as an important laboratory basis for excluding axSpA and supporting the diagnosis of DISH.

The patient exhibited symptoms such as lumbosacral pain and limited mobility, which bear some similarity to the inflammatory low back pain of axSpA, leading to the initial misdiagnosis of axSpA. However, the normal CRP result indicated the absence of significant systemic inflammatory responses in the patient, contradicting the pathological basis of “inflammatory pain” in axSpA. This prompted clinicians to reevaluate the diagnostic direction. Combined with imaging features such as “no sacroiliac joint inflammation” and “ossification of the anterior longitudinal ligament of the spine,” the final diagnosis of DISH was confirmed. It is evident that the normal CRP result broke the inertial thinking of “pain equating to inflammation,” providing objective laboratory evidence for differential diagnosis and avoiding misdiagnosis due to symptomatic similarity.

In the literature, numerous similar case reports highlight the misdiagnosis and clinical challenges associated with DISH. For instance, 1 report described a case of DISH misdiagnosed as axSpA,^[11]^ where the patient’s symptoms highly overlapped with those of axSpA, including spinal back pain, limited mobility, and spinal joint calcification on X-rays. Further examinations confirmed the diagnosis as DISH rather than an inflammatory spondyloarthropathy. This case is highly similar to ours, particularly in terms of significant symptomatic overlap. Additionally, other studies have noted that DISH may present distinct imaging features compared to typical axSpA. For example, the “calcified bridging” and extensive calcification of spinal ligaments commonly observed in DISH patients may be easily confused with the “bamboo spine” appearance of axSpA. Relatively, the ossification in DISH is usually more extensive and curved, rather than the typical segmental bony fusion seen in axSpA.

In summary, the initial diagnosis of axSpA was a transitional judgment resulting from overlapping symptoms, imaging findings, and imprecise application of diagnostic criteria. The final confirmation of DISH as the sole diagnosis through comprehensive assessment underscores the decisive role of imaging details (especially the presence or absence of sacroiliac joint inflammation) and laboratory tests in differential diagnosis.

## 5. Limitations

In exploring the issue of misdiagnosis between DISH and axSpA, this study has the following limitations: Restricted generalizability due to single-case analysis:The study was based solely on clinical data from a 67-year-old male patient, whose characteristics (age, gender, disease duration, and comorbidities such as osteoporosis) are specific and cannot fully represent the overall landscape of misdiagnosis between DISH and axSpA in diverse populations. In particular, DISH manifestations in young and middle-aged individuals may differ from those in elderly patients, and a single case cannot cover these variables, limiting the generalizability of the findings. Risk of information bias in retrospective research: case data relied on historical medical records, with missing details in some early examinations (e.g., specific descriptions of the first sacroiliac joint imaging), which may have hindered accurate tracking of the misdiagnosis trajectory. Additionally, recall bias in patients’ subjective symptom reporting and variations in examination standards across medical institutions could have compromised the accuracy of some clinical data. Limitations in the application of diagnostic criteria: the diagnosis of DISH in this study primarily relied on radiological criteria such as Resnick and Niwayama, which are more suitable for advanced cases and less sensitive in identifying early or atypical ossification. Despite multidisciplinary consultations, systematic validation of other potential differential diagnostic criteria (e.g., Utsinger criteria) was lacking, which may have affected the comprehensiveness of the diagnostic methodology.

Lack of long-term treatment follow-up data: the patient was discharged after 7 days of short-term treatment with symptom relief, and long-term follow-up data (e.g., rate of ossification progression, durability of drug efficacy, and incidence of complications) were unavailable. This prevented a full assessment of the specific impact of misdiagnosis on disease prognosis and hindered verification of the long-term effectiveness of the treatment strategy. Technical constraints in imaging analysis: the study did not quantitatively compare the efficacy of different imaging modalities (e.g., X-ray, CT, MRI) in differentiating DISH from axSpA, nor did it include quantitative analysis of ossification lesions (e.g., bone bridge length, calcification degree). This may have limited in-depth exploration of the differential value of imaging features.

These limitations suggest that future research should expand the sample size, include multi-center and multi-age cohort cases, adopt prospective designs with extended follow-up periods, and integrate quantitative imaging analysis and cross-validation of multiple diagnostic criteria. Such approaches would more comprehensively reveal the differential points and misdiagnosis mechanisms between these 2 diseases.

## 6. Implications

Based on the clinical insights and improvement suggestions, the following key points are proposed: thorough consideration of differential diagnosis:The diagnosis of DISH requires awareness of its similarities to other spinal conditions, particularly axial spondyloarthritis (axSpA) and degenerative spinal diseases. For patients with spinal calcification – especially middle-aged and elderly individuals – DISH should be prioritized as a critical differential diagnosis to avoid misclassification as inflammatory or degenerative disorders.

Enhanced recognition of imaging features: imaging plays a pivotal role in DISH diagnosis. Clinicians must deepen their understanding of DISH-specific radiological manifestations, including extensive calcification of spinal/pelvic ligaments and “bridging” ossification. In diagnostic workflows, imaging features should be carefully evaluated alongside clinical symptoms. For complex cases, CT or MRI should be used to clarify the scope and nature of lesions.

Integration of clinical manifestations and laboratory tests: DISH diagnosis should not rely solely on imaging but must integrate patient history, clinical symptoms, and laboratory data. While DISH typically lacks systemic inflammatory responses, mild elevations in chronic inflammatory markers (e.g., ESR, CRP) may occur. The efficacy of symptomatic treatment and the evolution of symptoms are also critical diagnostic clues.

For suspected DISH cases – particularly those with spinal calcification – comprehensive evaluation via multiple examination modalities is essential. Improved sensitivity for early diagnosis: early detection of DISH is clinically vital to avoid inappropriate treatment and delays. For atypical cases, heightened vigilance is required. Multidisciplinary collaboration should be employed to consider diverse diagnostic possibilities and deploy appropriate imaging/laboratory assessments.

This case and similar literature reports highlight the challenges of differentiating DISH from other spinal diseases (e.g., axSpA) due to overlapping clinical and imaging features. Clinicians must strengthen their ability to recognize DISH, particularly in patients with spinal calcification, by meticulously integrating medical history, clinical presentation, and diagnostic tests. Additionally, more precise imaging analysis and laboratory evaluation can enhance diagnostic accuracy. By improving awareness and prioritizing early diagnosis, misdiagnosis can be effectively reduced, ensuring timely and appropriate treatment for patients.

## Author contributions

**Formal analysis:** Xiuqing Luo.

**Writing – original draft:** Shiwei Yuan, Xuanhua Yu.

**Writing – review & editing:** Siyu Liu.
